# Novel allylated monocarbonyl analogs of curcumin induce mitotic arrest and apoptosis by reactive oxygen species-mediated endoplasmic reticulum stress and inhibition of STAT3

**DOI:** 10.18632/oncotarget.20924

**Published:** 2017-09-15

**Authors:** Vinothkumar Rajamanickam, Heping Zhu, Chen Feng, Xi Chen, Hailun Zheng, Xiaohong Xu, Qianqian Zhang, Peng Zou, Guodong He, Xuanxuan Dai, Xi Yang, Yi Wang, Zhiguo Liu, Guang Liang, Guilong Guo

**Affiliations:** ^1^ Chemical Biology Research Center, School of Pharmaceutical Sciences, Wenzhou Medical University, Wenzhou, Zhejiang 325035, P.R. China; ^2^ Department of Interventional Radiology, The Fifth Affiliated Hospital of Wenzhou Medical University, Lishui, Zhejiang, 323000, P.R. China; ^3^ Department of Anesthesiology, The First Affiliated Hospital of Wenzhou Medical University, Wenzhou, Zhejiang 325035, P.R. China; ^4^ Department of Surgical Oncology, The First Affiliated Hospital of Wenzhou Medical University, Wenzhou, Zhejiang 325035, P.R. China; ^5^ The Eye Hospital of Wenzhou Medical University, Wenzhou, Zhejiang 325027, P.R. China

**Keywords:** gastric cancer, curcumin analog, reactive oxygen species, ER stress, pSTAT3

## Abstract

Curcumin is a promising active compound from a natural source and is extensively being tested in clinical trials because of its bio-functional properties. However, poor bioavailability has hampered its clinical application. Numerous attempts have been made in our laboratory to discover analogs of curcumin with enhanced bioavailability and superior pharmacological activity. In this study, we have investigated a new series of allylated monocarbonyl analogs of curcumin (MAC) and tested their effect on gastric cancer cells. Our results show six MAC that selectively targeted cancer cell lines to inhibit growth and induce apoptosis. This activity was achieved by generation of reactive oxygen species (ROS) by MAC. We selected one effective MAC (CA10) for further investigation and show that CA10 inhibits cell growth by causing G2/M cell cycle arrest and induction of apoptotic death. CA10 induced ROS generation and subsequent activation of endoplasmic reticulum (ER) stress and inhibition of signal transducer and activator of transcription 3 (STAT3) phosphorylation, inhibits cancer cell proliferation. These anti-tumor activities of CA10 were confirmed in gastric cancer xenografts. CA10 induced ROS, activated the ER stress pathway and inhibited STAT3 phosphorylation and gastric xenografts tumor growth in mice. Our studies provide experimental evidence that MAC CA10 effectively targets gastric cancer in preclinical models by enhancing ROS and ROS-mediated signaling.

## INTRODUCTION

Medicinal herbs have served as an excellent resource for drug design and discovery. Curcumin is a naturally occurring hydrophobic polyphenolic compound which is isolated from the rhizome of the traditional herbal plant (*Curcuma longa Linn*). Curcumin has been used for centuries for treating various ailments due to its broad spectrum of pharmacological properties [1, 2]. Curcumin has also been extensively investigated for its anticancer activity against different cancers. Anti-proliferative and pro-apoptotic activities in cancer cells by curcumin have been demonstrated [3–5]. Furthermore, several clinical trials show that relatively high doses of curcumin (8 and 12 g/day) are relatively harmless. Despite the biological efficacy and safety profile of curcumin, it has not yet been approved as a therapeutic agent. This is because of pharmacokinetic limitations such as extensive metabolism and rapid elimination [6, 7]. For the past ten years, research from our laboratory has been actively involved in exploring various approaches to improve the bioavailability of curcumin. We have found that modification of the chemical structure of curcumin carries potential for effective drug discovery. In recent studies, we designed and synthesized a series of novel monocarbonyl analogs of curcumin (MAC) by deleting unstable moiety and modifying its substituents at different positions [8, 9]. These alterations have produced promising pharmacokinetic profiles of MAC compared to that of curcumin [8, 9]. In addition, experimental evidence also confirms that MAC may have other advantages, including a) improved potency, solubility and stability, b) enhanced selective toxicity, c) reduced side effects, and d) improved pharmacokinetic properties. Furthermore, we showed that MAC are highly effective against a panel of tumor cell lines [10]. Although the mechanism of the anti-tumor activity of MAC is still not clear.

Oxidative stress resulting from increased production of reactive oxygen species (ROS) has been implicated in several diseases including cancer. High rate of metabolic activities in cancer cells increases ROS levels and the activation of ROS-sensitive oncogenic pathways through the activation of transcription factors, such as nuclear factor-κB (NF-κB), activator protein-1 (AP-1), hypoxia-inducible factor-1α (HIF-1α) and signal transducer and activator of transcription 3 (STAT3) [11–13]. Even though moderate oxidative stress is beneficial to the cancer cells to attain uncontrolled proliferation, angiogenesis and metastasis [14], exorbitant increases in ROS make cancer cells vulnerable and can enhance apoptotic cell death [15, 16]. Hence, regulation of ROS levels and ROS-mediated signaling remains a crucial point for intervention and the discovery of new anti-cancer drugs.

An allyl group has been found in many natural products, such as Honokiol, Obavatol, Ferrearin C and Simonols A. These compounds also show potent anticancer activity through enhancing cytotoxicity, elevation of ROS and ROS-mediated signaling pathways [17–21]. In addition, analogs of Honikinols with allyl groups exhibit promising anti-proliferative and anti-angiogenic effects in various cancer cell lines [22]. Similarly, curcumin-based allylated MAC synthesized in our laboratory by stabilizing the central β-diketone linker with a single carbonyl group and modifying its structure by allylic substituents, showed potent pharmacological activity [23–25]. To build on our previous studies, we have explored potential anti-cancer effects and the underlying mechanism of the newly discovered allylated MAC here. Our studies demonstrate that allylated MAC significantly suppressed cancer cell growth in culture and in animal models through ROS generation and the ability to interfere with multiple pathways regulating growth and apoptosis. We also show that dual allylic compound CA10 may be an excellent candidate for further studies as a possible treatment for gastric cancer.

## RESULTS

### Cytotoxic screening of allylated MAC on various gastrointestinal cell lines

First, we investigated whether allylated MAC alter the viability or have cytotoxic effects on cells in culture. We have performed MTT tetrazolium reduction assay following exposure of cancer cells (gastric cancer, BGC823 and SGC-7901; colon cancer, HT-29, HCT-116 and SW-480) and normal gastric epithelial cells (GES-1) to all 20 allylated MAC at a concentration of 20 μM for 24 h. We show that MAC produce a pronounced inhibition of tumor cell growth (Table 1). Among these allylated MAC, CA1, CA6, CA10, CA13, CA15 and CA20 showed selective cytotoxicity in cancer cell lines with minimal inhibition of the normal epithelial cells. In addition, we also found notable solubility and stability profile of these compounds (Supplementary Figure 1).

**Table 1:** Chemical structures and cytotoxic potential of all synthetic compounds 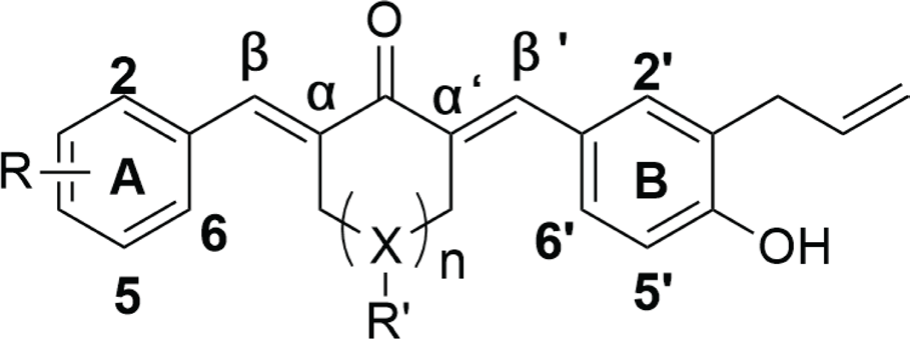

Comp.	X	R	Rʹ	n		% Of growth inhibition					
					Con. μM	BGC-823	SGC-7901	HT-29	HCT-116	SW-480	GES-1
CA1	N	3-allyl, 4-OH	-CH_2_CH_3_	1	20	60.39 ± 0.62	66.08 ± 8.88	52.93 ± 2.67	50.22 ± 7.62	71.26 ± 3.35	22.79 ± 3.04
CA2	N	3-allyl, 4-OH	-CH_2_CH_2_CH_3_	1	20	63.05 ± 8.70	79.77 ± 0.90	67.01 ± 6.79	60.57 ± 0.98	76.01 ± 6.31	60.88 ± 0.41
CA3	N	3-allyl, 4-OH	-CCH(CH_3_)_2_	1	20	63.62 ± 2.34	70.28 ± 8.15	66.97 ± 8.19	50.85 ± 2.57	71.11 ± 3.13	50.66 ± 6.74
CA4	N	3-allyl, 4-OH	-Cyclopropyl	1	20	67.06 ± 0.40	77.75 ± 1.98	74.27 ± 0.79	44.18 ± 6.18	81.02 ± 5.36	63.35 ± 2.67
CA5	N	3-allyl, 4-OH	-SO_2_-Benzene	1	20	63.34 ± 5.52	82.02 ± 2.61	71.22 ± 1.99	43.04 ± 4.24	77.06 ± 3.79	63.79 ± 2.33
CA6	N	3-allyl, 4-OH	-Benzyl	1	20	65.83 ± 0.52	83.71 ± 6.29	79.72 ± 3.11	72.58 ± 2.72	79.09 ± 4.20	22.55 ± 6.08
CA7	C	3-allyl, 4-OH	N/A^a^	1	20	71.25 ± 3.07	86.38 ± 2.74	79.46 ± 4.10	66.99 ± 4.92	71.67 ± 1.15	66.69 ± 1.45
CA8	O	3-allyl, 4-OH	N/A^a^	1	20	71.18 ± 1.07	82.31 ± 2.16	71.07 ± 2.60	38.83 ± 4.23	79.32 ± 4.37	27.00 ± 3.40
CA9	S	3-allyl, 4-OH	N/A^a^	1	20	69.26 ± 3.13	84.89 ± 3.58	75.70 ± 2.18	67.28 ± 5.03	77.96 ± 5.69	58.69 ± 4.41
CA10	C	3-allyl, 4-OH	N/A^a^	0	20	67.48 ± 1.54	84.61 ± 3.73	77.15 ± 4.86	75.37 ± 4.52	77.40 ± 7.84	23.40 ± 7.48
CA11	N	4-Methylpiperazine	-Cyclopropyl	1	20	61.33 ± 0.48	61.01 ± 4.97	63.34 ± 1.31	52.05 ± 1.91	49.55 ± 6.47	45.56 ± 0.39
CA12	N	4-Morpholino	-Cyclopropyl	1	20	40.09 ± 4.65	52.47 ± 5.54	56.60 ± 4.22	70.99 ± 3.12	33.80 ± 1.98	39.95 ± 3.29
CA13	N	4-Pyrrolyl	-Cyclopropyl	1	20	64.85 ± 6.79	82.77 ± 0.13	61.75 ± 1.93	61.12 ± 3.67	43.24 ± 6.45	37.48 ± 1.96
CA14	N	4-N,N-Diethyl	-Cyclopropyl	1	20	40.42 ± 5.70	80.49 ± 1.22	59.93 ± 8.01	52.10 ± 9.05	56.96 ± 3.57	47.90 ± 8.09
CA15	N	4-N,N-Dimethyl	-Cyclopropyl	1	20	50.63 ± 2.49	50.55 ± 4.30	42.58 ± 8.71	43.17 ± 8.26	36.28 ± 1.70	23.77 ± 4.79
CA16	N	2,4,5-OCH_3_	-Cyclopropyl	1	20	60.69 ± 5.64	60.03 ± 7.35	67.97 ± 1.39	60.11 ± 2.02	35.69 ± 5.91	41.34 ± 5.22
CA17	N	3,4-OCH_3_	-Cyclopropyl	1	20	36.97 ± 4.35	73.70 ± 1.26	55.04 ± 5.09	78.06 ± 064	64.72 ± 4.65	61.94 ± 1.41
CA18	N	2,4,6-OCH_3_	-Cyclopropyl	1	20	57.96 ± 0.01	61.31 ± 4.02	63.05 ± 4.20	74.79 ± 1.25	56.88 ± 3.82	57.79 ± 3.58
CA19	N	2,4-OCH_3_	-Cyclopropyl	1	20	59.16 ± 3.67	76.59 ± 5.18	62.59 ± 7.39	78.11 ± 4.72	60.39 ± 3.10	62.00 ± 4.58
CA20	N	4-OC(CH_3_)_3_	-Cyclopropyl	1	20	56.79 ± 0.81	85.87 ± 5.39	71.16 ± 0.60	73.62 ± 4.23	61.45 ± 6.10	25.80 ± 1.86

N/A^a^-Means no substituents. The cytotoxic effects of allylated curcumin analogs in human gastric and colon cancer cells and normal gastric epithelial cells was determined by MTT assay and the IC_50_ values are presented as the mean ± SD of three independent experiment with four duplicates.

### Structure-activity relationship (SAR)

We next aimed to describe the structural activity relationship (SAR) of the allylated MAC. We have observed that all ten structurally symmetrical compounds (CA1 to CA10) showed significant growth inhibition rate in BGC-823 cells and this inhibition rate were ranged from 60.39% to 71.25%. Interestingly, we have observed only three of the asymmetrical compounds with different substituted group such as: methylpiperazine (CA11), pyrrolyl (CA13), 2,4,5-OCH_3_ (CA16), showed 61.33%, 64.85% and 60.69% inhibition rate. Other structurally asymmetrical compounds with a morpholino group (CA12), N,N-diethyl (CA14), N,N-dimethyl (CA15) and different substituted OCH_3_ group (CA17 to CA20) exhibited poor growth inhibition in BGC-823 cells, which ranged from 36.97% to 59.16%.

We noted different level of toxicity when we assayed SAR information from another gastric cancer cell line, SGC-7901. All compounds show high growth inhibition rate from 60.03% to 86.38% in SGC-7901 except two compounds with a morpholino group (CA12) and N,N-dimethyl (CA15). These two compounds only showed 52.47% and 50.55% of growth inhibition. Symmetrical compounds with a different substituted group for the piperidine ketone part including SO_2_-Benzene (CA5), benzyl group (CA6) or other ketone such as cyclohexanone (CA7), Tetrahydro-4H-pyran-4-One (CA8), Tetrahydro thiopyran-4-one (CA9), cyclopentanone (CA10), as well as asymmetrical compounds with pyrrolyl (CA13), N,N-diethyl (CA14) or OCH(CH_3_)_3_ (CA20) exhibited excellent cytotoxicity in SGC-7901 (exceeding 80% inhibition).

Further, SAR screening of allyllated MAC on three different colon cancer cell lines were analyzed. Interestingly, we found that all ten symmetrical compounds with different ketone exhibit strong growth inhibition rate on SW-480 cells, which were ranged from 71.11% to 81.02%. However, only three of ten asymmetrical compounds with substitution of two OCH_3_ or OCH(CH_3_)_3_ exhibited moderate cytotoxicity in SW-480 cells: CA17 (64.72%), CA19 (60.39) and CA20 (61.45). Typically, other asymmetrical compounds containing a hydrophilic group (CA11 to CA15) or three OCH_3_ groups (CA16 and CA18) showed inferior activity, which have shown only 33.80% to 56.96% of growth inhibition. Furthermore, when assessed in HT-29, most of these compounds revealed great cytotoxic activity except CA1, CA12, CA14, CA15 and CA17, which showed less than 60% of growth inhibition.

Analysis of HCT-116 colon cancer cells showed that symmetrical compounds with benzyl group (CA6), cyclopentanone (CA10) or asymmetrical compounds with morpholino group (CA12), OCH(CH_3_)_3_ (CA20) as well as different quantities of OCH_3_ group (CA17 to CA19) exhibit better cytotoxicity with an inhibition rate of 70%. Among symmetrical compounds containing ethyl (CA1), CH(CH_3_)_2_ (CA3), cyclopropyl (CA4), SO_2_-benzene (CA5), tetrahydro-4H-pyran-4-one (CA8) or asymmetrical compounds with methylpiperazine (CA11), N,N-diethyl (CA14), N,N-dimethyl (CA15) groups, appear to weakly inhibit growth in HCT-116 at a rate of 55%. Some compounds such as CA2, CA7, CA9, CA13 and CA16, exhibited intermediate cytotoxicity (60.11% to 67.28%) in HCT-116 cell line. In normal gastric epithelial cells (GES-1), only compounds CA2-CA5, CA7, CA9, and CA17-CA19 inhibited growth greater than 60%.

**Figure 1:**
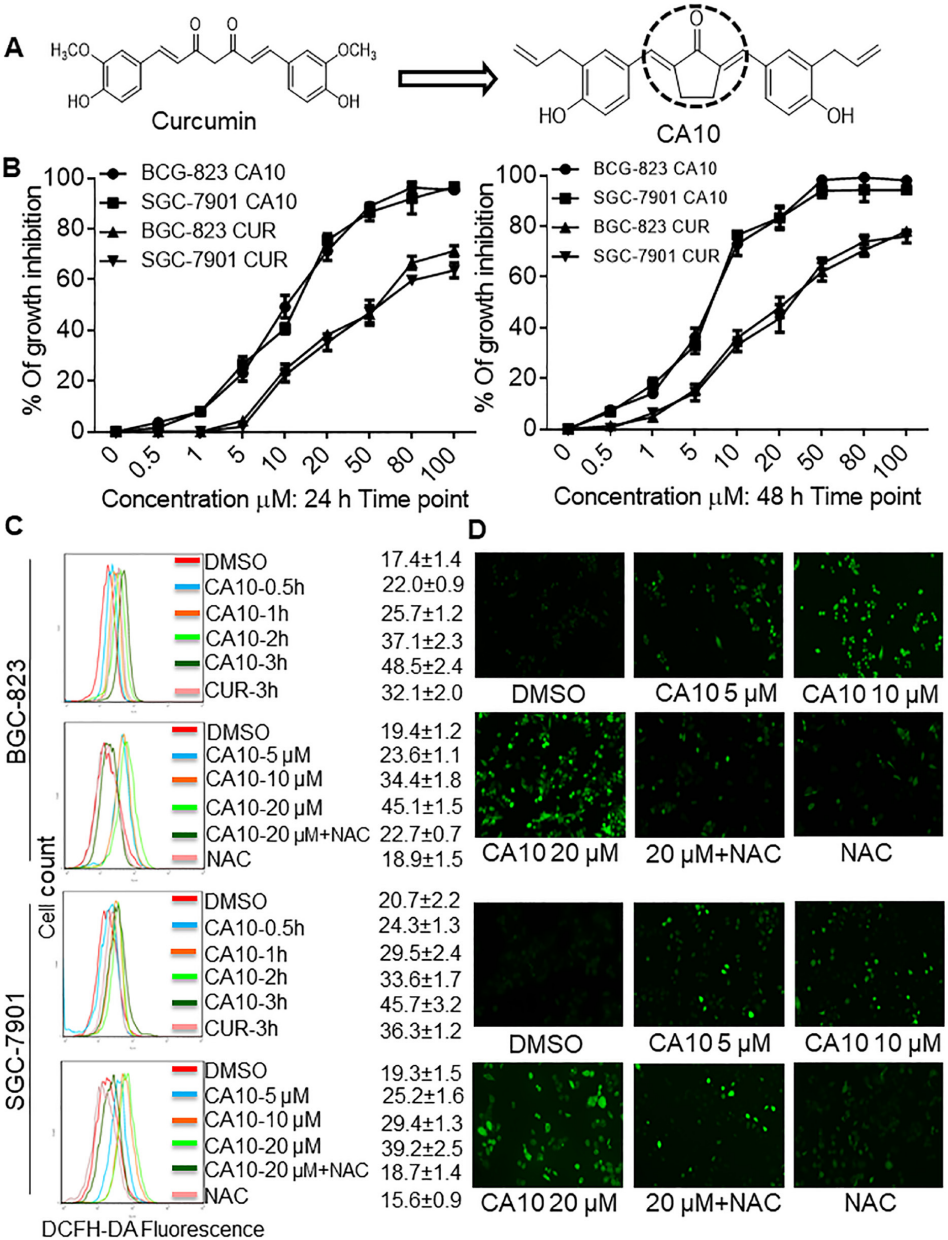
CA10 exhibits selective cancer cell cytotoxicity and induces ROS (**A**) The structure of parental compound curcumin and its analog CA10. (**B**) The effect of curcumin and CA10 on human gastric cancer cell viability. Cell viability was determined by the MTT assay and the IC_50_ values are presented as the mean ± SD of three independent experiment with four duplicates. (**C**) Flow cytometric analysis of intracellular ROS generation by CA10 at various time (0.5, 1, 2 and 3 h) and doses (5, 10 and 20 μM) in both BGC-823 and SGC-7901 cells. ROS levels were measured by DCFH-DA. Cells were pre-treated with 5 mM NAC for 2 h before exposure to CA10. The data are represented as the mean ± SD of three independent experiment. (**D**) Fluorescence microscopic images showing intracellular ROS levels (green) induced by CA10 at different doses.

As illustrated in Table 1, most of the allylated MAC inhibited the growth of gastric and colonic cancer cells. However, CA10 and CA6 compounds showed significant growth inhibition in all cancer cell lines, while exhibiting markedly lower cytotoxic effect in normal gastric epithelial cells. Other compounds showed differential ability to inhibit cancer cell growth. For example, CA8 and CA20 inhibited most cell lines but were not as effective as CA10 in inhibiting the growth of HCT-116, and SW-480 and BGC-823 cells, respectively. While CA15, which contains N,N-dimethyl group, showed modest inhibitory activity towards the five cancer cell lines. Finally, structurally symmetrical compounds containing two allyl groups exhibited pronounced cytotoxicity compared to asymmetrical compounds with only one allyl group. Based on this screening, we selected CA10 for further pharmacological analysis to investigate its anti-cancer effects. The chemical structure of mother compound curcumin and CA10 was shown in Figure 1A.

### Dose-dependent cytotoxicity and comparison of CA10 to curcumin

We performed a dose-response study to obtain IC_50_ values for CA10. We selected two gastric cancer cell lines (BGC-823 and SGC-7901) and exposed these cells to various concentration of CA10 (0.5, 1, 5, 10, 20 50 80 and 100 μM) for 24 h or 48 h. As shown in Figure 1B, treatment with CA10 significantly inhibited tumor cell viability and these effects were observed in a dose-dependent manner. In addition, we have noticed that IC_50_ values at the dose of 13.02 ± 2.48 μM in BGC-823 and 14.97 ± 1.72 μM in SGC-7901 at 24 h time point. In comparison, curcumin required a higher concentration (IC_50_, BGC-823: 54.38 ± 3.92 μM and SGC-7901: 62.17 ± 2.08 μM) to induce 50% growth inhibition (IC_50_). In addition, prolonged exposer (48 h) of curcumin and CA10 to gastric cancer cells exhibits minimal IC_50_ values (CA10: BGC-823 7.14 ± 1.69 μM and SGC-7901 6.91 ± 0.43 μM; Curcumin: BGC-823 38.25 ± 1.02 μM and SGC-7901 36.88 ± 3.17 μM) indicates time dependent cytotoxic effect. Based on the strong inhibition of gastric cancer cells proliferation, possible cellular and molecular mechanisms underlying CA10-induced growth inhibition in gastric cancer cells were investigated.

### CA10, triggers ROS-dependent G2/M cell cycle arrest and growth inhibition

Previous studies have shown that curcumin elevates intracellular ROS and mitochondrial collapse, which leads to apoptotic cell death in numerous cancer cells [26, 27]. In addition, numerous natural and synthetic allyl compounds stimulate ROS production in malignant cancer cells [21, 22]. These findings prompted us to investigate whether ROS elevation was involved in the cytotoxic effect of these allylated MAC. We measured ROS generation by utilizing 2,7-diacetyl dichlorofluorescein (DCFH-DA) which gets hydrolyzed by the cellular esterases to DCFH and is then oxidized by ROS to yield fluorescent dichlorofluorescein (DCF). Our results show that CA10 and other selective allyl compounds, indeed, increased ROS levels in SGC-7901 cells (Supplementary Figure 2A) was consistent with observed cytotoxicity. To confirm that ROS mediated cytotoxicity in this system, we pretreated the cells with N-acetyl cysteine (NAC) for 2 h prior to exposing the cells to CA10. Pretreatment of cells with NAC reduced the level of CA10-induced growth inhibition (Supplementary Figure 2B), suggesting that CA10-induced ROS mediates cytotoxicity.

**Figure 2:**
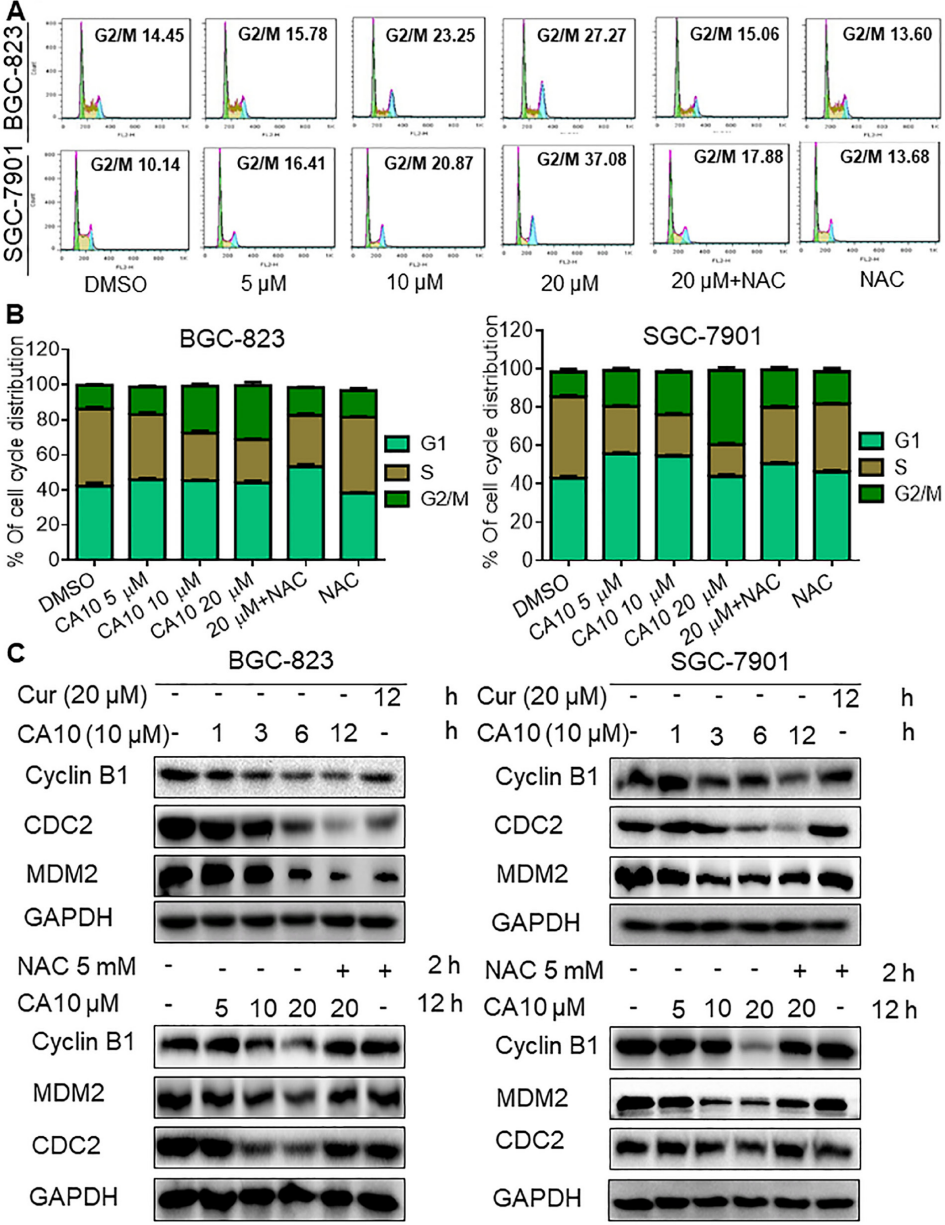
CA10 promotes ROS-dependent G2/M cell cycle arrest (**A**) BGC-823 and SGC-7901 cells were treated with varying concentrations of CA10 for 16 h, and the cell cycle distribution was analyzed by flow cytometry. (**B**) Histogram illustrates the percentage of cell distribution at different cell cycle phases. The results were obtained from FACS analysis of three independent of experiments. (**C**) Immunoblot analysis of G2/M regulators including CDC-2, MDM-2 and Cyclin B1 at various time (1, 3, 6 and 12 h) and doses (5, 10 and 20 M) of CA10 exposure. Three independent experiments were performed and GAPDH was used as internal control.

To confirm the role of ROS in CA-induced gastric cancer cell toxicity, we performed time- and dose-response experiments utilizing both BGC-823 and SGC-7901 cells. Flow cytometric and fluorescence microscopic detection of DCF showed time- and dose-dependent increases in ROS levels by CA10 (Figure 2C and 2D). ROS generation by CA10 was noticed at an earlier time in both BGC-823 and SGC-7901 compared to the parental compound curcumin. Cells pre-treated with 5 mM NAC for 2 h prior to incubation with CA10 (at 5, 10 and 20 μM) for 3 h showed that NAC significantly reduced intracellular ROS accumulation induced by CA10 to levels comparable to that of control cells (Figure 1C and 1D). Generally, accumulation of ROS by exogenous agent could alters the levels of oxidative stress markers. Malondialdehyde (MDA), a lipid peroxidation by product, is a marker of cellular oxidative damage. In order to confirm if ROS induced by CA10, exert further oxidative stress in cancer cells, we measured MDA a by-product generated from lipid peroxidation. Indeed, CA10 treatment caused an increased MDA level in SGC-7901 cells. Further, enhanced MDA production was noticed at the dose of 20 μM. On the other hand, we observed marginally decreased level of total GSH in comparison to control (Supplementary Figure 2E and 2F). The alteration in the total GSH and MDA levels in cancer cells could be due to over utilization of anti-oxidant system to counteract ROS insult under extreme ROS condition induced by CA10. These findings clearly show the involvement of ROS in CA10-induced viability changes.

To understand the mechanisms, we first examined whether the ROS-mediated growth inhibition entails cell cycle deregulation. We observed increased population of cells in the G2/M cell cycle phase upon CA10 exposure for 16 h. Greatest accumulation in G2/M phase was noted with 20 μM CA10. Furthermore, pre-treatment of cells with NAC attenuated G2/M arrest (Figure 2A and 2B). Next, we assessed the level of several key proteins (cell division cycle protein 2 (CDC-2)/Cyclin B1 complex, and murine double minute 2 (MDM-2)) linked to G2/M cell cycle transition to confirm our flow cytometric data. We show that CA10 treatment reduces the expression of CDC2, Cyclin B1 and MDM2 (Figure 2C) confirming G2/M cycle arrest in gastric cancer cells. Moreover, inhibition of ROS with NAC normalized the level of these G2/M cycle proteins (Figure 2C, lower panels).

**Figure 3:**
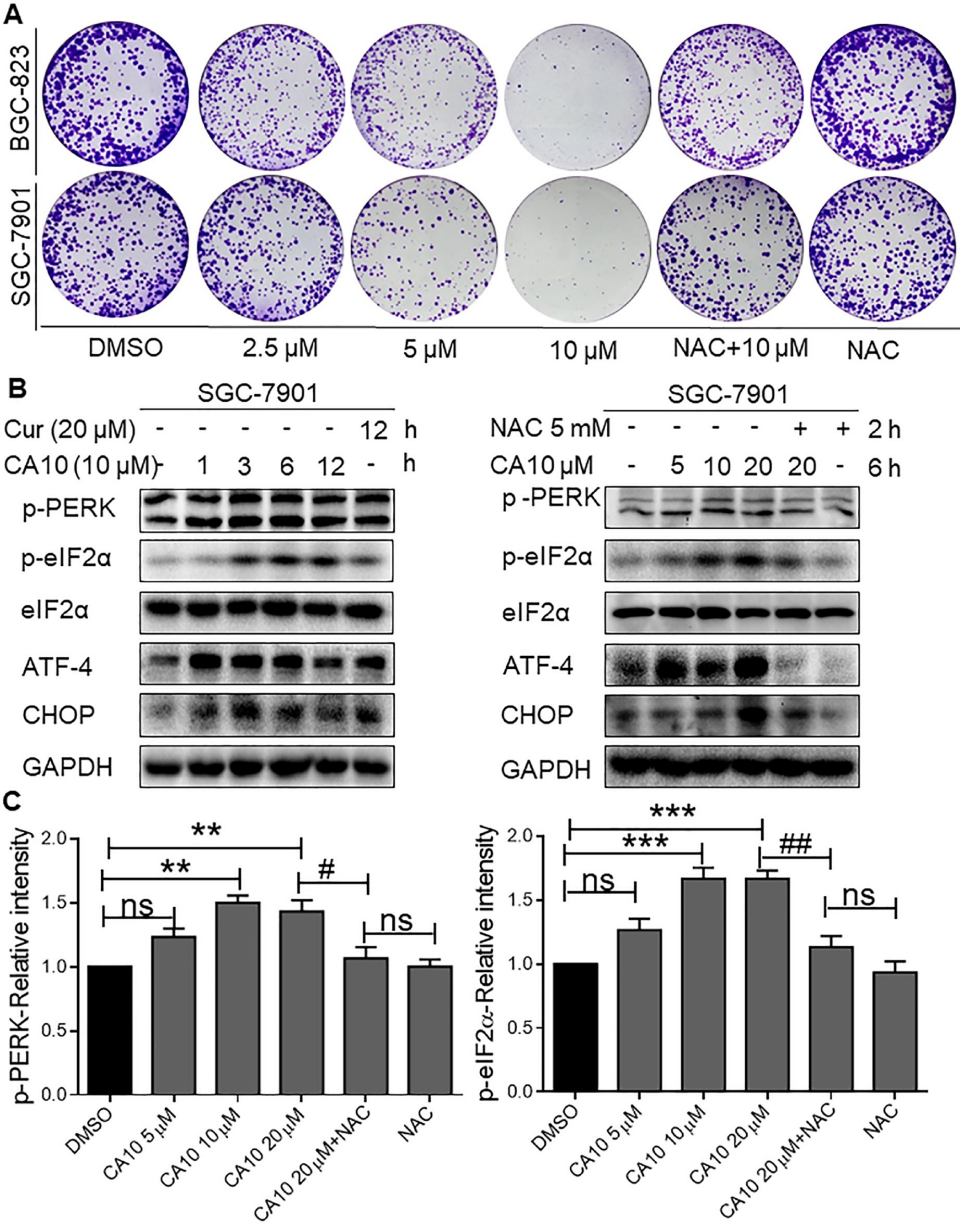
CA10 induces ROS-mediated growth inhibition and ER stress response (**A**) BGC-823 and SGC-7901 cells were exposed to various concentrations of CA10 (2.5, 5 or 10 μM) or DMSO (vehicle control) for 24 h. Media was then replaced with fresh medium and culture continued for 7 days. Colonies were stained by crystal violet and then photographed. Three independent experiments were executed to view colonies formation. (**B**) BGC-823 and SGC-7901 cells were treated with CA10 for various time (0.5, 1, 2 and 3 h) and doses (5, 10 and 20 μM). Protein levels of p-PERK, p-EIF2α, ATF4 and CHOP were determined by western blotting. Representative of three independent experiments is shown. GAPDH and EIF2α were used as internal control. (**C**) Histogram data indicates the p-PERK and p-eIF2α expression by image J analysis of three independent experiments (^*^P < 0.01, ^***^P < 0.001 compared to the control group; ^#^*p* < 0.05 ^##^*p* < 0.01 compared to the treatment group (CA10 20 μM).

We next performed a colony formation assay and show reduced number of BGC-823 and SGC-7901 colonies upon treatment of cells with CA10 for 24 h. Greatest response was observed with the highest concentration of CA10 compared to 2.5 or 5 μM. Using this platform, we also show that NAC pretreatment diminished the ability of CA10 to inhibit tumor cell colony formation (Figure 3A). These results confirm that CA10 induces ROS-dependent cell cycle arrest and growth inhibition.

### MAC CA10 induces ROS-dependent ER stress response

Experimental evidence shown that induction of ROS generation can trigger apoptotic cell death by stimulating the ER stress pathway [9]. In this study, we have observed that CA10 elevates ROS levels in gastric cancer cells in a short time point (Figure 1C). Therefore, we aimed to determine whether the anti-tumor effect of CA10 on gastric cancer cells was associated with ROS-ER stress pathways. To do this, SGC-7901 cells were treated with CA10 at the dose of 10 μM for 1, 3, 6 and 12 h. Interestingly, protein blot analyses showed increased expression of ER specific proteins such as phosphorylated protein kinase-like endoplasmic reticulum kinase (p-PERK), phosphorylated eukaryotic initiation factor 2 (p-eIF2α), activating transcription factor-4 (ATF-4) and CCAAT/enhancer-binding protein homologous protein (CHOP) in the SGC-7901 cells (Figure 3B). Furthermore, prominent expression on ER key proteins was noticed at 3–6 h time point, which indicates short time is sufficient to activate ER stress responses in SGC-7901 by CA10. Additionally, to determine whether ROS involvement in CA10 induced ER stress responses, ROS scavenging experiment was conducted by utilizing NAC. Interestingly, in presence of NAC we observed decreased expression of ER stress key proteins (p-PERK, p-EIF2α, ATF4, and CHOP) upon CA10 treated, whereas CA10 alone treatment shown increased expression of ER stress responses (Figure 3B right panels and 3C) in SGC-7901 cells, which shows CA10 induced ROS generation is involved in activation of ER stress pathway.

### ROS mediated p-STAT3 inhibition and induction of apoptosis in SGC-7901 cells

**Figure 4:**
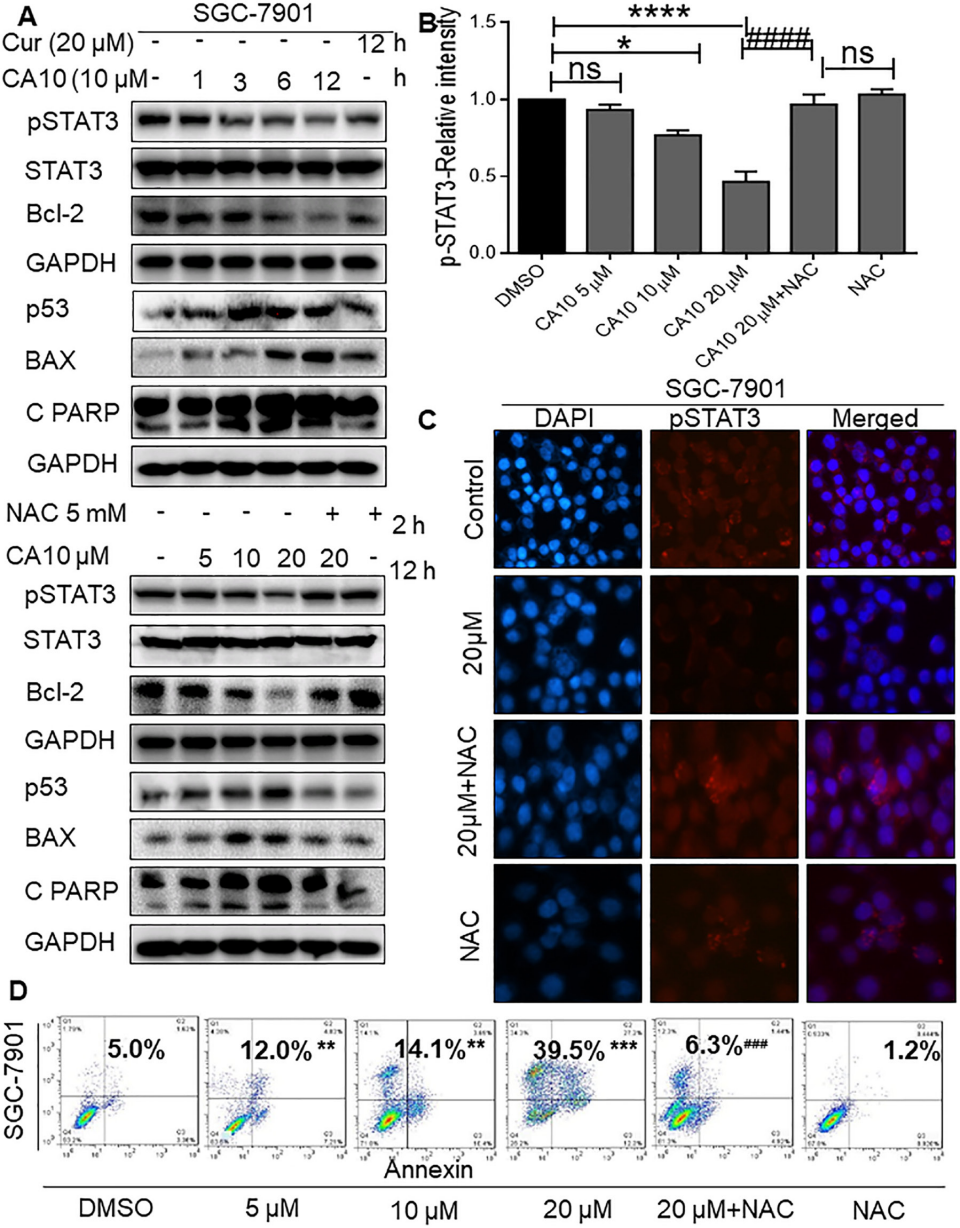
Elevated ROS inhibits p-STAT3 and induces apoptosis in SGC-7901 cells (**A**) SGC-7901 cells were treated with CA10 for various time (0.5, 1, 2 and 3 h) and doses (5, 10 and 20 μM). Protein levels of p-STAT3, Bcl2, Bax, p53 and Cleaved PARP were determined by western blotting. GAPDH and STAT3 were used as internal control. Three independent experiments were performed. (**B**) Histogram data indicates the pSTAT3 expression by image J analysis of three independent experiments (^*^P < 0.05, ^****^P < 0.0001 compared to the control group; ^####^*p* < 0.0001 compared to the treatment group (CA10 20 μM). (**C**) Fluorescence cell staining for p-STAT3 showing week nuclear reactivity for p-STAT3 upon CA10 treatment. Images shown at 20× magnification. (**D**) Flow cytometric analysis of apoptotic cell death showing both early and late apoptosis induced by CA10 in SGC-7901 cells. The statistical difference between groups are indicated with respective symbols (^**^P < 0.01, ^***^P < 0.001 compared to the control group; ^###^*p* < 0.001 compared to compared to the treatment group (CA10 20 μM).

Next, we investigated the underlying mechanisms of the anti-cancer effect by CA10. Studies have demonstrated that STAT3 is aberrantly activated in a number of human cancers and plays a crucial role in tumor initiation and progression by regulating Bcl2 family members [28–30]. Therefore, we determined whether CA10-induced ROS, and subsequent ER stress and growth inhibition is linked to alteration of STAT3. In this study, we observed high basal levels of p-STAT3 and altered expression of apoptotic proteins such as tumor protein 53 (p53), B-cell lymphoma-2 (Bcl-2), Bcl-2 associated X protein (Bax) and cleaved poly (ADP-ribosyl) polymerase (PARP) in gastric cancer cells (SGC-7901) as expected. However, treatment with CA10 various time points (1, 3, 6 and 12 h) decreased the protein levels of Bcl-2 and p-STAT3 but not total STAT3 proteins. Not surprisingly, constitutively active p53, Bax and cleaved-PARP were also observed upon CA10 treatment (Figure 4A). Collectively these results suggested that the p-STAT3 and Bcl-2 was involved in the execution of apoptotic cell death in CA10 treated SGC-7901 gastric cancer cells.

Previously, we found that ROS elevation was an important mediator of the beneficial effects of CA10 in terms of causing ER stress activation and inhibiting cancer cell growth. To dissect out the signaling mechanisms, we examined whether ROS elevation was associated with the inhibition of p-STAT3 and altered expression of apoptotic responses. Cells were treated with various doses of CA10 for 12 h with or without 2 h pretreatment of 5 mM NAC. Our results showed that pretreatment of cells with NAC prevented CA10 mediated changes in the expression of p-STAT3, as well as in the expression of apoptosis-related proteins (Figure 4A-lower panels, 4B and 4C). In addition, microscopic analysis of cells and flow cytometric analysis of Annexin V/PI stained cells confirmed induction of apoptosis in SGC-7901 cells by CA10. We noted altered morphology of cancer cells (cell shrinkage, loss of membrane integrity, nuclear swelling and condensed chromatin) (Supplementary Figure 2C and 2D) and accumulation of cells at early and late apoptotic stages (Figure 4D). Taken together, these results confirmed that CA10 amplifies ROS generation, leading to growth inhibition, and induction of apoptosis possibly through inhibiting oncogenic p-STAT3.

### CA10 inhibited SGC-7901 xenograft tumor growth by inducing apoptosis through ROS-mediated inhibition on p-STAT3

**Figure 5:**
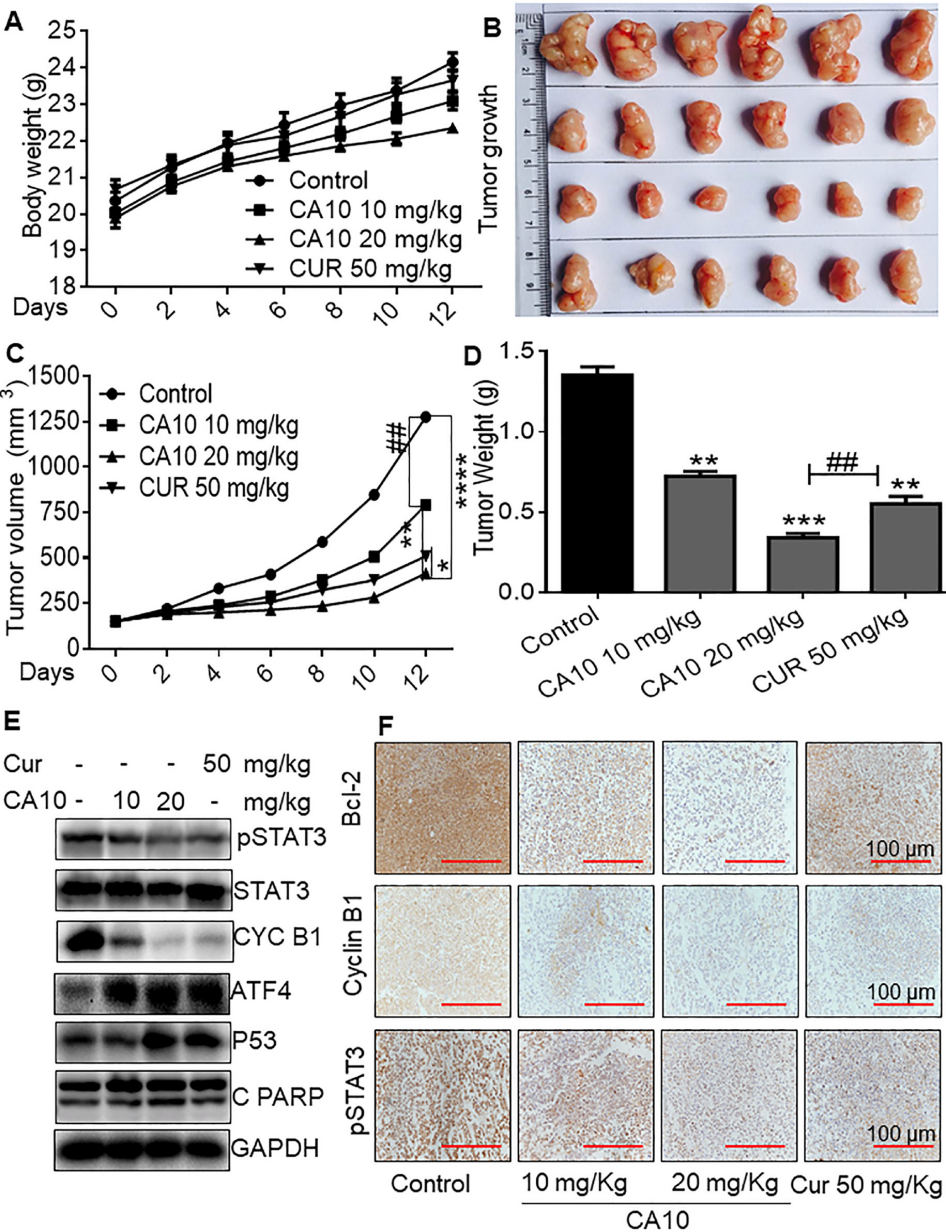
CA10 inhibited SGC-7901 xenograft growth by inhibition on p-STAT3 (**A**) Body weight of mice showing no significant changes in various experimental groups. (**B**) Photographs of extracted SGC-7901 xenograft tumors from various experimental groups. (**C**) Quantification of tumor volume at various time points including 0, 2, 4, 6, 8, 10 and 12 days. (**D**) Graph illustrating tumor weight from various experimental groups (^*^P < 0.05, ^**^P < 0.01, ^***^P < 0.001, ^****^P < 0.0001 compared to the control group; ^##^*P* < 0.01 compared to curcumin group, all data presented as the mean ± SD of six mice in each group). (**E**) Western blot analysis of p-STAT3, cyclin B1, ATF4, p53 and cleaved PARP protein expression in respective xenograft tumors. (**F**) Immunohistochemical staining for p-STAT3, Bcl-2 and Cyclin B1 expression in xenograft tumor tissues. Control mice showing strong expression of p-STAT3, Bcl-2 and Cyclin B1. However, mice treated with CA10 and curcumin show moderate to week levels of p-STAT3, Bcl-2 and Cyclin B1 immunoreactivity.

Based on the promising inhibitory effects of CA10 on tumor cell growth *in vitro*, next we aimed to confirm whether CA10 would exhibit anti-tumor effects *in vivo*. To do this, we produced SGC-7901 xenograft tumors. Once tumors had reached a volume of 100–200 mm^3^, mice were treated with intraperitoneal (ip) injection of CA10 (10 and 20 mg/kg), curcumin (50 mg/kg), or vehicle alone, once a day for 12 days. As shown in Figure 5A, there was no change in the body weight of mice in the different treatment groups (0–12 days). We observed a significant reduction in the tumor weight and volume when mice were treated with CA10 or curcumin (Figure 5B–5D). Furthermore, CA10 at the dose of 20 mg/kg showed a greater inhibition effect on tumor growth compared to CA10 low dose (10 mg/kg) or curcumin at 50 mg/kg. Western blot analyses of the extracted tumor tissues showed significantly decreased levels of p-STAT3, and increased levels of ATF4, p53 and cleaved-PARP (Figure 5E). We also observed decreased levels of G2/mitotic-specific cyclin, cyclin-B1 (Figure 5E). These results were validated by immunohistochemical staining of tumor tissues showing decreased immunoreactivity for p-STAT3, Bcl-2 and cyclin B1 in mice treated with CA10 and curcumin (Figure 5F). These results indicates that CA10 at the dose of 20 mg/kg exhibits more pronounced effect by suppressing tumor growth, compare to low doses of CA10 and curcumin.

**Figure 6:**
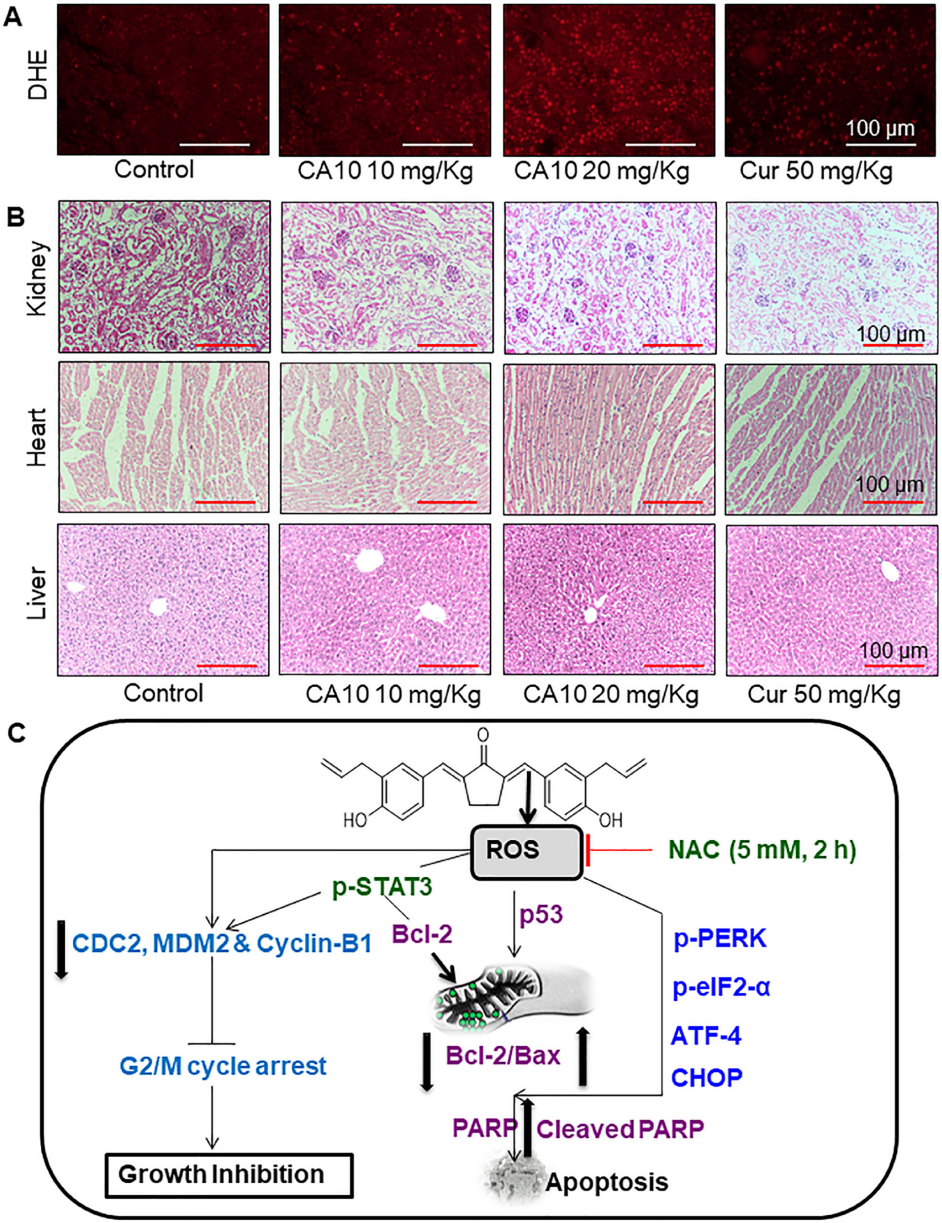
CA10 inhibits gastric cancer tumor xenograft growth by increasing ROS (**A**) Fluorescence images showing DHE in tumor specimens. DHE indicates induction of ROS by CA10 and curcumin treatment in xenogarft tumor tissues. (**B**) Brighfield images of H&E stained sections of heart, liver and kidney from the 4 experimental groups of mice. Histological analysis shows no evidence of significant pathological alterations. (**C**) Schematic illustration of the underlying mechanism of CA10-induced anti-cancer activity.

As our *in vitro* studies showed that ROS increases may be upstream of CA10-induced anti-cancer effects, we determined the levels of generation in our xenograft model. Our results in tumor specimens show increased CA10-induced ROS levels as evidenced by increased DHE positive cells (red fluorescence) (Figure 6A). These data show that ROS-mediated p-STAT3 inhibition and potential ER stress mediates the apoptotic signals in CA10 treated SGC-7901 xenograft tumors. These beneficial effects of CA10 were observed without overt toxicity in vital organs including the heart, liver and kidney tissues (Figure 6B). In summary, our *in vivo* study revealed that the pharmacological action of CA10 were tumor-specific and fared well compared to the parent compound, curcumin.

## DISCUSSION

Despite of the continuous improvements in the technical features of cancer detection and management, gastric cancer patients is still thought to be predominantly incurable due to its metastatic potential and drug resistance [31]. Therefore, searching predictable target based therapeutic agent against GC, has recently attracted much attention. A large body of evidence suggests that structurally modified analog compounds are effective than its parental compound by inhibiting cancer cell growth [32–34]. Besides, previous studies from our lab also have shown that analogs exerts marked therapeutic advantages in pre-clinical settings. Importantly, we reported that similar like parental compound, analogs compounds exhibits wide spectrum of pharmacological activity by targeting multitude signaling pathways [35, 36]. Herein, we found that a novel allylated MAC selectively inhibits gastrointestinal cancer cell growth. In addition, we noted elevated ROS levels and ROS dependent growth inhibition in cancer cells by allylated MAC. These results suggested that the allylated MAC is a potent and safe candidate for induction of therapeutic ROS and new drug discovery. Further studies reveals, effective compound CA10 can prompts dose- and time-dependent toxicity and ROS. Here, we have revealed that minimal concentration of CA10 sufficient for induction of 50% toxicity, but parental compound curcumin exerts similar toxicity at higher concentration. Further, we have seen high selective cytotoxicity in various cancer cells. Henceforth, possible cellular and molecular mechanisms underlying CA10 induced growth inhibition in gastric cancer cells were further examined.

It is well know that excessive ROS production is associated with the induction of cell death and differentiation, fascinated in therapeutic strategy [37]. Numerous small molecules used to treat various malignant tumors have been shown to stimulate high level of ROS [33, 38]. Our results also concurrent with them, shown ROS mediated cytotoxicity and loss of cancer cell integrity. Destruction of cell cycle progression is an appreciated target for cancer treatment. In this present study, we have observed decreased invasive colonies and G2/M phase cell cycle arrest upon CA10 treatment, which indicates that CA10 could reduce the aggressiveness of gastric cancer cells. Indeed, G2/M transition is regulated by the cell division cycle protein 2 (CDC-2)/Cyclin B1 complex [39], and murine double minute 2 (MDM-2) is a negative regulator of p21, which causes cell cycle arrest in the G2/M phase [40]. In consistent with above statement, CA10 treatment reduced the protein expression of CDC2, Cyclin B1 and MDM2, this further emphasizes that CA10 can inhibit the aggressive potential of gastric cancer cells through G2/M cycle arrest. Moreover, inhibition of ROS with NAC as a ROS quencher eliminated the CA10 induced G2/M cycle arrest, which suggested that ROS stimulation as a crucial feature of allylated MAC, CA10 mediated anti-cancer effect.

A large body of evidence indicates that therapy-induced oxidative stress may activate endoplasmic reticulum (ER) stress-related apoptosis [41, 42]. Furthermore, cellular stresses and cytotoxic conditions lead to ER stress via activation of unfolded protein response (UPR) signaling cascade [43]. The UPR pathway involves activation of three ER transmembrane proteins including PERK, protein kinase and site specific endonuclease (IRE1) and activating transcription factor 6 (ATF6). Of these proteins, PERK activation appears to be pivotal for the signaling cascade [44]. PERK leads to phosphorylation of α subunit of eukaryotic initiation factor 2 (eIF2α) which causes global repression of translation [45, 46]. However, one of the proteins for which translation is increased is activating transcription factor 4 (ATF4). ATF4 translation and activity subsequently results in the induction of C/EBP-homologous protein (CHOP) and promotion of ER stress-induced apoptosis [47]. Therefore, we examined key ER stress specific proteins in SGC-7901 cells to determine whether CA10-induced ROS participates in ER stress activation. We first treated SGC-7901 cells with 10 μM of CA10 for 1, 3, 6 and 12 h. Protein blot analyses showed that 3–6 h exposure is sufficient to induce ER specific proteins: phosphorylated PERK (p-PERK), p-EIF2α, ATF4, and CHOP in the SGC-7901 cells. Another interesting observation of the current study is that CA10 induced PERK axis is blocked by NAC pre-treatment, which shows that the anti-cancer effects of CA10 are ROS-dependent.

A recent study has shown that induction of ER stress modulates signal transducer and activator of transcription 3 (STAT3) activity and causes apoptosis in ovarian cancer cells [48]. Interestingly, our results have shown inhibition of STAT3 phosphorylation upon CA10 treatment, observed in dose and time-dependent manner in SGC-7901 cells. Remarkably, besides its decrease (p-STAT3), anti-apoptotic protein Bcl-2 was also decreased. In addition to these observational changes in p-STAT3/Bcl-2, obviously increased expression of p53, pro-apoptotic Bax and cleavage of PARP denotes apoptosis inducing potential of CA10. Moreover, we showed that pharmacological inhibition of p-STAT3 was completely abrogated by NAC pre-treatment, suggesting that ROS generation may be the upstream regulator in p-STAT3 inhibition. Thus, we considered that the ROS dependent down-regulation of Bcl-2 and G2/M cell cycle regulators (MDM2, Cyclin B1 and CDC-2) was likely to be liked with the ability of CA10 to prompt apoptosis in SGC-2901 cells. Since, activated STAT3 protects tumor cells from apoptosis and promote cell growth by regulating multiple genes associated with cell proliferation and anti-apoptotic proteins such as surviving cyclin D1,c-Myc, Fas, Mcl-1, Bcl-xL, and Bcl-2 [49, 50]. Study has also shown that the suppression of constitutively active STAT3 in ROS dependent manner can cause growth inhibition and apoptosis in lung cancer cells as well as in xenograft models [51]. Interestingly, curcumin has been reported to decrease STAT3 phosphorylation in colon and lung cancer-derived cells in a dose-dependent manner [52, 53] It is concluded that pharmacological inhibition of p-STAT3 by CA10, delayed its oncogenic nature, and thereby induces apoptotic cell death in gastric cancer cells.

Moreover, the *in vivo* SGC-7901 xenograft findings demonstrated that, CA10 at different doses significantly inhibited tumor growth without altering the body weight of mice. Similar to our *in vitro* findings, *in vivo* data also provides evidence that feasibility of apoptotic death by inducing ROS and promising alteration in the p53, ER stress and p-STAT3 signaling pathways. Furthermore, CA10 is considered to be safe as the concentration of 20 mg showed no toxic side effects in vital organs. Of the two tested doses, the high dose of 20 mg/kg showed the maximum beneficial effect by suppressing tumor growth. This could possibly be due to the following reasons: a) the low dose may not be sufficient to suppress tumor growth b) the high dose may resulted high ROS and toxic effect tumor site, which in turn caused significant inhibition on tumor growth. Therefore, these collective evidence suggested that CA10 could be safe and pharmacologically effective than parental compound curcumin.

In conclusion, our study demonstrates that CA10 effectively reduced growth of gastric cancer by increasing ROS generation both *in vitro* and *in vivo*. We further show that CA10 evoked G2/M cell cycle deregulation and apoptosis in cancer cell lines. These effects were mediated through an induction of ROS and activation of the ER stress pathway and inhibition of p-STAT3 (Figure 6C). However, further molecular investigations in preclinical and clinical settings are needed to evaluate the selective anticancer potential of CA10 and to substantiate this MAC as an effective therapeutic candidate for gastric cancer.

## MATERIALS AND METHODS

### Chemistry

**Figure 7:**
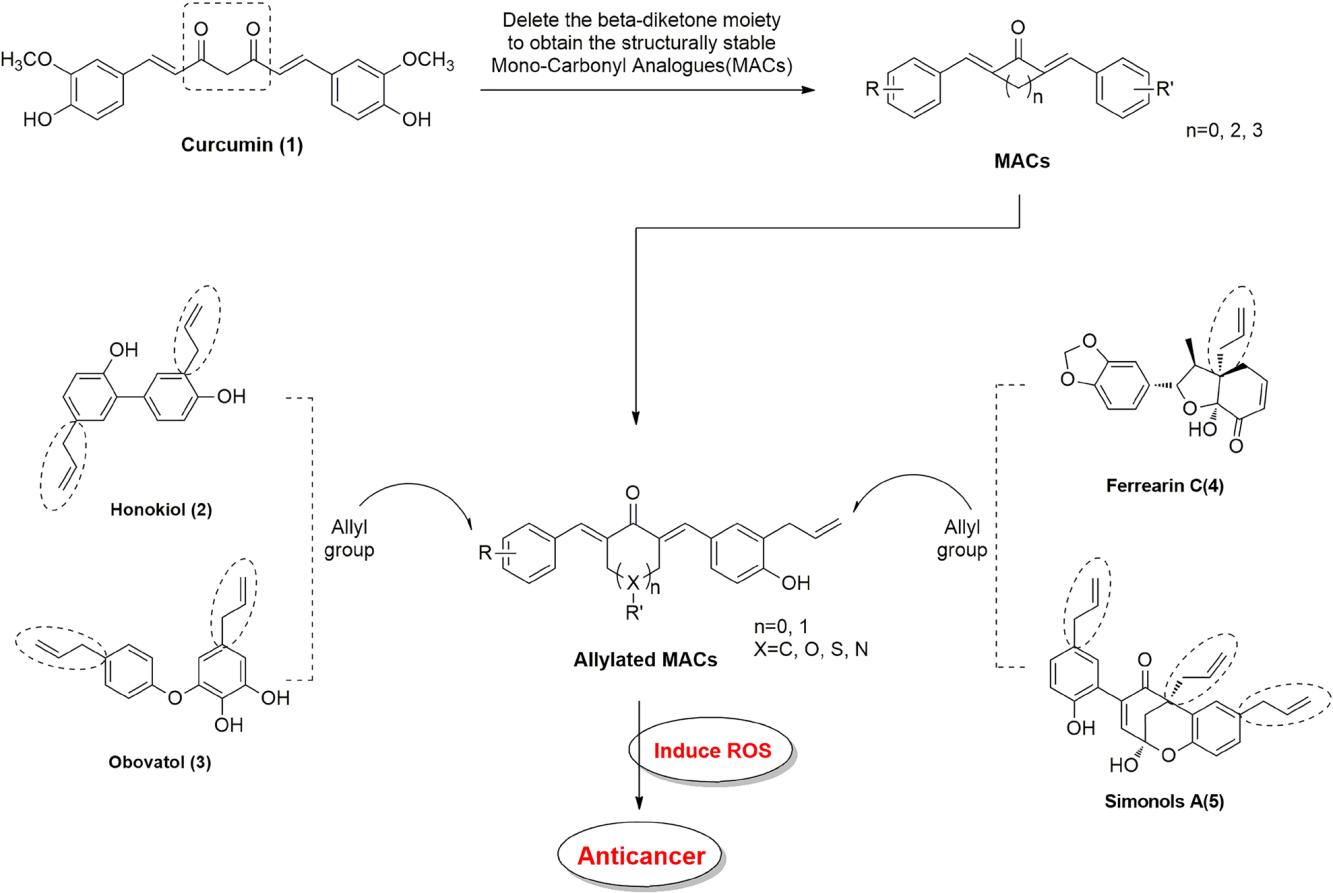
Structural design of allylated mono-carbonyl analogs of curcumin (MAC)

A series of allylic monocarbonyl analogs of curcumin (MAC) were synthesized based on the mother compound, curcumin, by direct aldol condensation of substituted benzaldehyde with acetone or cyclopentanone in alkaline media. We then identified their structural difference by using MS and NMR analysis. The detailed synthesis protocol for allylated MAC is described in our previous publications [23–25]. In this report, Figure 7 exemplify the step by step synthetic route for allylated MAC. Before using in biological studies, all allylated MAC were recrystallized from CHCl_3_/EtOH, and HPLC was used to determine the purity. The results demonstrated that all compounds had a purity of ≥ 95%. The molecular structures of all target compounds is presented in Table 1.

### Chemicals and reagents

Curcumin was purchased from Sigma Chemical Co. (St. Louis, MO). Apoptosis and cell cycle detection kits (Annexin V/Propidium Iodide (PI) and PI/RNase) were purchased from BD Biosciences (Franklin Lakes, NJ). Assay kits for reactive oxygen species (2,7-Dichlorodihydrofluorescein diacetate, DCFH-DA) and oxidative stress markers (malondialdehyde, MDA; total GSH and DAPI sating solution) were obtained from Beyotime Biotech (Nantong, China). Antibodies against mouse double minute 2 (MDM-2), cyclin-dependent kinase 1 (CDC2), Cyclin B1, p53, B-cell lymphoma 2 (Bcl-2), Bcl2-associted protein x (Bax), cleaved form of poly (ADP-ribose) polymerase (PARP), phosphorylated protein kinase-like endoplasmic reticulum kinase (p-PERK), GAPDH, and horseradish/ fluorephore-conjugated secondary antibodies were purchased from Santa Cruz Biotechnology (Santa Cruz, CA, USA). Antibodies against CCAAT/enhancer-binding protein homologous protein (CHOP), activating transcription factor-4 (ATF-4), eukaryotic initiation factor 2 (eIF2α), signal transducer and activator of transcription 3 (STAT3), and phosphorylated STAT3 (p-STAT3) were purchased from Cell Signaling Technology (Danvers, MA).

### Cell lines and maintenance

Established human gastric cancer cell lines (BGC-823, SGC-7901), colon cancer cell lines (HT-29, HCT-116 and SW-480), and human normal gastric epithelial cell line (GES-1) were obtained from the Cell Bank of Shanghai Institutes for Biological Sciences (Shanghai, China). All cells were maintained in RPMI-1640 media (Gibco, Life Technology) supplemented with 10% heat-inactivated fetal bovine serum (FBS) (Gibco) and 1% antibiotic solution (100 units/mL penicillin and 100 μg/mL streptomycin; Gibco). Media was changed every 2–3 days and subculture was performed at 70–80% confluency. All experiments were performed during exponential growth phase of cells.

### Assessment of cell toxicity

Cells were plated at a density of 8 × 10^3^ cells/well in 96-well plates and allowed to attach overnight. Stock solutions of allylated MAC and curcumin were prepared in DMSO and diluted in culture media to obtain appropriate concentration. Cells were treated for 24 h and 48 h, following which, 20 μL of 5 mg/ml MTT reagent was added. Purple formazan product was dissolved by the addition of 100 μL DMSO to each well. Absorbance was then monitored at 490 nm using a plate reader (Molecular Device Spectra MAX190, USA). Percentage of cytotoxicity was determined using the following formula: Growth inhibition (%) = (OD of control−OD of test/OD of control×100%).

### Determination of reactive oxygen species (ROS) generation

Generation of reactive oxygen species (ROS) was determined using 2, 7-dichlorofluorescein diacetate (DCFH-DA). Briefly, BGC-823 and SGC-7901 were plated at 3 Î 10^5^ cells on 60-mm dishes, allowed to attach overnight, and exposed to compounds at various doses (5, 10 and 20 μM) and for different time points (0.5, 1, 2, 3 h). In some experiments, cells were pretreated with 5 mM NAC for 2 h prior to challenging with compound CA10. Cells were stained with 1 μL of DCFH-DA (10 μM) at 37°C for 30 min. Then, cells were collected and fluorescence was measured using FACS Calibur flow cytometer (BD Biosciences, CA). Fluorescence microscopy (Nikon, Japan) was also used to examine intracellular ROS levels and to take images (Nikon, Japan).

### Clonogenic assay

The BGC-823 and SGC-7901 cells were seeded at 1000 cells/well in 6 well plates and allowed to attach overnight. Cells were then exposed to various concentrations (2.5, 5 and 10 μM) of CA10 for 24 h in presence and absence of NAC (5 mM). Following treatments, media was changed to fresh culture media and cultures were followed for 7 days. The experiment was terminated by washing the colonies with PBS and fixation with 4% methanol at room temperature for 10 min. Colonies were then stained with Crystal violet for 10 min and counted.

### Cell cycle analysis

BGC-823 and SGC-7901 were seeded at 3 × 10^5^ density in 6-well plates and treated with various doses of CA10 for 14 h, with or without 2 h pretreatment with 5 mM NAC. After treatment, cells were harvested, fixed in ice cold 75% ethanol. DNA was labeled with 0.5 mL of PI/Rnase Staining buffer (BD Bioscience) at a final concentration of 0.05 mg/mL for 20 min in the dark at 4°C. Distribution of cell cycle phases was analyzed on BD FACSCalibur flow cytometer (BD Biosciences, USA).

### Annexin V-FITC/PI apoptosis determination

Apoptosis was determined using Annexin V-FITC/PI apoptosis detection kit. BGC-823 and SGC-7901 cells were seeded at a density of 3×10^5^ cells per well in 6-well plates and allowed to attach overnight. Cells were then treated with 5, 10 and 20 μM of CA10 for 24 h time point, with or without pretreatment of 2 h with 5 mM NAC. Cells were harvested, re-suspended in 500 μL binding buffer, and stained with 3 μL of FITC-conjugated anti-Annexin V for 10 min and 1 μL Propidium iodide (PI) for 5 min. Apoptosis was analyzed with FACSCalibur flow cytometer and the results were analyzed using FlowJo software.

### Determination of morphological features of apoptosis

SGC-7901 cells were seeded at 3 × 10^5^ cells in 60-mm dishes, allowed to attach overnight and then exposed to compound 20 μM CA10 for 14 h, with or without 2 h pretreatment with 5 mM NAC. Cells were then examined by phase contrast microscopy or fluorescence microscopy following staining with DAPI.

### Estimation of Malondialdehyde (MDA) and total GSH in cancer cells

The treated cells were harvested with RIPA buffer at 4°C. Cell lysates were then centrifuged at 12,000 × g for 10 min at 4°C to collect the supernatant. Protein content was determined by the Bradford protein assay kit. Equal amounts of proteins were used for malondialdehyde (MDA) and total GSH assay (Beyotime Institute of Biotechnology, Nantong, China). MDA and total GSH levels were detected by measuring absorbance respectively at 532 nm and 412 nm using a multimode microplate reader (Molecular Devices, SpectraMax 190, USA

### Western blot analysis

Protein samples were prepared from cells following treatments or tumor tissues. Protein concentration was determined by Bradford protein assay kit (Bio-Rad, Hercules, CA). Samples were separated on precast 8%-12% SDS polyacrylamide gels. After electrophoresis, proteins were transferred to poly vinylidene difluoride (PVDF) membrane. The membranes were blocked with 5% non-fat milk in tris-buffered saline containing 0.1% Tween 20 (TBST) for 90 min at room temperature, then primary antibodies (1:1000) in TBST were added and membranes incubated overnight at 4°C. After washing with TBST, horseradish peroxidase (HRP)-conjugated secondary antibodies (1:3000) were added for 1 hour at room temperature. After additional washing with TBST, enhanced chemiluminescence detection system (ECL, GE Healthcare) was used. Membranes were probed for GAPDH as a loading control. The density of the immunoreactive bands was analyzed using Image J (National Institute of Health, MD).

### *In vivo* mouse xenograft model

All animal experiments complied with the Wenzhou Medical University's Policy on the Care and Use of Laboratory Animals. Protocols for animal studies were approved by the Wenzhou Medical University Animal Policy and Welfare Committee (Approved documents: 2016/201). Athymic BALB/c nu/nu female mice weighing 20–22 g (6 weeks old) were purchased from Vital River Laboratories (Beijing, China). Animals were housed (6 animals/gage) at a constant room temperature with a 12/12-hr light/dark cycle and fed a standard rodent diet and water *ad libitum*. SGC-7901 cells were injected subcutaneously into the right flank of each mice at 1 × 10^6^ cells in 0.1 mL PBS. Once tumors reached a volume of 100–200 mm^3^, mice were randomly divided into 4 groups. These groups included mice treated with 10 mg/kg CA10, mice treated with 20 mg/kg CA10, mice treated 50 mg/kg curcumin, and control mice receiving vehicle alone. All treatments were carried out with intraperitoneal injections. The body weight and tumor volumes were monitored and determined by measuring length (l) and width (w) and calculated as tumor volume (V = 0.5 × l × w2) at the indicated time points. At the end of treatment period, mice were sacrificed and the tumors were removed and weighed for histological analyses and proteins measurement studies.

### Determination of tissue superoxide production

Portion of tumor tissues extracted from mice were immediately embedded in OCT compound and cut into 5 μm-thick sections. Tissue sections were then incubated with dihydroethidium (DHE, 10 mM) in PBS at 37°C for 30 min in dark. DHE is oxidized upon reaction with superoxide to ethidium bromide, which then binds to DNA. Red fluorescence from ethidium bromide was viewed and captured using a fluorescence microscope (λ_ex_ 490 nm, λ_em_610 nm, 20× magnification; Nikon, Japan).

### Histological analyses

Portions of heart, liver, kidney and tumor tissues from mice were fixed in 4% paraformaldehyde solution for 1 week at room temperature. Specimens were then dehydrated by passing through graded ethanol, cleared in xylene, and embedded in paraffin wax. Tissue blocks were sectioned at 5 μm thickness and used for H&E and immunohistochemical staining. Tumor tissue sections were stained with indicated primary antibodies for overnight followed by secondary antibodies for 2 h. Diaminobenzidine (DAB) was used for detection. The slides were counterstained with hematoxylin for 30 seconds and mounted. For histologic analysis of heart, kidney and liver tissue sections, we performed routine H&E staining and viewed the sections under a light microscope (20× magnification, Nikon, Japan).

### Statistical analysis

All experiments were repeated at least 3 times. Values from quantitative experiments are expressed as the mean ± standard deviation (SD). Significance was determined by Student's *t*-test and two-way analysis of variance (ANOVA) using GraphPad Pro 5.0 (GraphPad, San Diego, CA). Differences were considered to be significant at *P* < 0.05.

## SUPPLEMENTARY MATERIALS FIGURES



## Abbreviations

ATF4: Activating transcription factor-4
Bax: B-cell lymphoma-2 (Bcl-2) associated X protein
Bcl-2: B-cell lymphoma-2
CDC-2: Cell division cycle protein 2
CHOP: CCAAT-enhancer-binding protein (C/EBP), homologous protein
C/EBP: CCAAT-enhancer-binding protein
DCF: Dichlorofluorescein
DCFH-DA: 2, 7-dichlorofluorescein diacetate
ER: Endoplasmic reticulum
FBS: Fetal bovine serum
FITC: Fluorescein isothiocyanate
GC: Gastric cancer
GSH: Glutathione
MDM-2: Murine double minute 2
NAC: n-Acetyl cysteine
p53: Tumor protein 53
PARP: Poly (ADP-ribosyl) polymerase (PARP)
p-EIF2α: Phosphorylated eukaryotic initiation factor 2α
PI: Propidium iodide
p-PERK: Phosphorylated protein kinase RNA-like endoplasmic reticulum kinase
ROS: Reactive oxygen species
STAT3: Signal transducer and activator of transcription 3
IRE1: Protein kinase and site specific endonuclease
ATF6: Activating transcription factor-6
MAC: Monocarbonyl analogs of curcumin
MDA: Malondialdehyde

## References

[1] Quiles JL, Mesa MD, Ramírez-Tortosa CL, Aguilera CM, Battino M, Gil A, Ramírez-Tortosa MC (2002). Curcuma longa extract supplementation reduces oxidative stress and attenuates aortic fatty streak development in rabbits. Arterioscler Thromb Vasc Biol.

[2] Goel A, Jhurani S, Aggarwal BB (2008). Multi-targeted therapy by curcumin: how spicy is it?. Mol Nutr Food Res.

[3] Das L, Vinayak M (2012). Anti-carcinogenic action of curcumin by activation of antioxidant defence system and inhibition of NF-κB signaling in lymphoma-bearing mice. Biosci Rep.

[4] Zhang L, Cheng X, Gao Y, Bao J, Guan H, Lu R, Yu H, Xu Q, Sun Y (2016). Induction of ROS-independent DNA damage by curcumin leads to G2/M cell cycle arrest and apoptosis in human papillary thyroid carcinoma BCPAP cells. Food Funct.

[5] Wang L, Chen X, Du Z, Li G, Chen M, Chen X, Liang G, Chen T (2017). Curcumin suppresses gastric tumor cell growth via ROS-mediated DNA polymerase γ depletion disrupting cellular bioenergetics. J Exp Clin Cancer Res.

[6] Anand P, Kunnumakkara AB, Newman RA, Aggarwal BB (2007). Bioavailability of curcumin: problems and promises. Mol Pharm.

[7] Klickovic U, Doberer D, Gouya G, Aschauer S, Weisshaar S, Storka A, Bilban M, Wolzt M (2014). Human pharmacokinetics of high dose oral curcumin and its effect on heme oxygenase-1 expression in healthy male subjects. Biomed Res Int.

[8] Liang G, Li X, Chen L, Yang S, Wu X, Studer E, Gurley E, Hylemon PB, Ye F, Li Y, Zhou H (2008). Synthesis and anti-inflammatory activities of mono-carbonyl analogues of curcumin. Bioorg Med Chem Lett.

[9] Zou P, Zhang J, Xia Y, Kanchana K, Guo G, Chen W, Huang Y, Wang Z, Yang S, Liang G (2015). ROS generation mediates the anti-cancer effects of WZ35 via activating JNK and ER stress apoptotic pathways in gastric cancer. Oncotarget.

[10] Liang G, Shao L, Wang Y, Zhao C, Chu Y, Xiao J, Zhao Y, Li X, Yang S (2009). Exploration and synthesis of curcumin analogues with improved structural stability both *in vitro* and *in vivo* as cytotoxic agents. Bioorg Med Chem.

[11] Waris G, Ahsan H (2006). Reactive oxygen species: role in the development of cancer and various chronic conditions. J Carcinog.

[12] Muthumani P, Alagarsamy K, Dhandayuthapani S, Venkatesan T, Rathinavelu A (2014). Pro-angiogenic effects of MDM2 through HIF-1α and NF-κB mediated mechanisms in LNCaP prostate cancer cells. Mol Biol Rep.

[13] Rad E, Dodd K, Thomas L, Upadhyaya M, Tee A (2015). STAT3 and HIF1α Signaling Drives Oncogenic Cellular Phenotypes in Malignant Peripheral Nerve Sheath Tumors. Mol Cancer Res.

[14] Wu WS (2006). The signaling mechanism of ROS in tumor progression. Cancer Metastasis Rev.

[15] Trachootham D, Alexandre J, Huang P (2009). Targeting cancer cells by ROS-mediated mechanisms: a radical therapeutic approach?. Nat Rev Drug Discov.

[16] Nogueira V, Hay N (2013). Molecular pathways: reactive oxygen species homeostasis in cancer cells and implications for cancer therapy. Clin Cancer Res.

[17] Lee SK, Kim HN, Kang YR, Lee CW, Kim HM, Han DC, Shin J, Bae K, Kwon BM (2008). Obovatol inhibits colorectal cancer growth by inhibiting tumor cell proliferation and inducing apoptosis. Bioorg Med Chem.

[18] Hayama T, Tabata K, Uchiyama T, Fujimoto Y, Suzuki T (2011). Ferrearin C induces apoptosis via heme oxygenase-1 (HO-1) induction in neuroblastoma. J Nat Med.

[19] Yin PJ, Wang JS, Wei DD, Zhang Y, Wang PR, Wang XB, Kong LY (2013). Simonols A and B, two novel sesqui-neolignans from the fruits of Illicium simonsii. Fitoterapia.

[20] Gao DQ, Qian S, Ju T (2016). Anticancer activity of Honokiol against lymphoid malignant cells via activation of ROS-JNK and attenuation of Nrf2 and NF-κB. J BUON.

[21] Lin CJ, Chen TL, Tseng YY, Wu GJ, Hsieh MH, Lin YW, Chen RM (2016). Honokiol induces autophagic cell death in malignant glioma through reactive oxygen species-mediated regulation of the p53/PI3K/Akt/mTOR signaling pathway. Toxicol Appl Pharmacol.

[22] Ma L, Chen J, Wang X, Liang X, Luo Y, Zhu W, Wang T, Peng M, Li S, Jie S, Peng A, Wei Y, Chen L (2011). Structural modification of honokiol, a biphenyl occurring in Magnolia officinalis: the evaluation of honokiol analogues as inhibitors of angiogenesis and for their cytotoxicity and structure-activity relationship. J Med Chem.

[23] Liu Z, Tang L, Zou P, Zhang Y, Wang Z, Fang Q, Jiang L, Chen G, Xu Z, Zhang H, Liang G (2014). Synthesis and biological evaluation of allylated and prenylated mono-carbonyl analogs of curcumin as anti-inflammatory agents. Eur J Med Chem.

[24] Qiu C, Hu Y, Wu K, Yang K, Wang N, Ma Y, Zhu H, Zhang Y, Zhou Y, Chen C, Li S, Fu L, Zhang X (2016). Synthesis and biological evaluation of allylated mono-carbonyl analogues of curcumin (MACs) as anti-cancer agents for cholangiocarcinoma. Bioorg Med Chem Lett.

[25] Zhu H, Xu T, Qiu C, Wu B, Zhang Y, Chen L, Xia Q, Li C, Zhou B, Liu Z, Liang G (2016). Synthesis and optimization of novel allylated mono-carbonyl analogs of curcumin (MACs) act as potent anti-inflammatory agents against LPS-induced acute lung injury (ALI) in rats. Eur J Med Chem.

[26] Khan MA, Gahlot S, Majumdar S (2012). Oxidative stress induced by curcumin promotes the death of cutaneous T-cell lymphoma (HuT-78) by disrupting the function of several molecular targets. Mol Cancer Ther.

[27] Chen Q, Wang Y, Xu K, Lu G, Ying Z, Wu L, Zhan J, Fang R, Wu Y, Zhou J (2010). Curcumin induces apoptosis in human lung adenocarcinoma A549 cells through a reactive oxygen species-dependent mitochondrial signaling pathway. Oncol Rep.

[28] Ji K, Zhang M, Chu Q, Gan Y, Ren H, Zhang L, Wang L, Li X, Wang W (2016). The Role of p-STAT3 as a Prognostic and Clinico pathological Marker in Colorectal Cancer: A Systematic Review and Meta-Analysis. PLoS One.

[29] Bhattacharya S, Ray RM, Johnson LR (2005). STAT3-mediated transcription of Bcl-2, Mcl-1 and c-IAP2 prevents apoptosis in polyamine-depleted cells. Biochem J.

[30] Xu J, Lin H, Li G, Sun Y, Shi L, Ma WL, Chen J, Cai X, Chang C (2017). Sorafenib with ASC-J9® synergistically suppresses the HCC progression via altering the pSTAT3-CCL2/Bcl2 signals. Int J Cancer.

[31] Zhang D, Fan D (2007). Multidrug resistance in gastric cancer: recent research advances and ongoing therapeutic challenges. Expert Rev Anticancer Ther.

[32] Wang W, VanAlstyne PC, Irons KA, Chen S, Stewart JW, Birt DF (2004). Individual and interactive effects of apigenin analogs on G2/M cell-cycle arrest in human colon carcinoma cell lines. Nutr Cancer.

[33] Sivagami G, Vinothkumar R, Bernini R, Preethy CP, Riyasdeen A, Akbarsha MA, Menon VP, Nalini N (2012). Role of hesperetin (a natural flavonoid) and its analogue on apoptosis in HT-29 human colon adenocarcinoma cell line--a comparative study. Food Chem Toxicol.

[34] Du C, Dong MH, Ren YJ, Jin L, Xu C (2016). Design, synthesis and antibreast cancer MCF-7 cells biological evaluation of heterocyclic analogs of resveratrol. J Asian Nat Prod Res.

[35] Wu J, Ji J, Weng B, Qiu P, Kanchana K, Wei T, Wang Y, Cai Y, Li X, Liang G (2014). Discovery of novel non-ATP competitive FGFR1 inhibitors and evaluation of their anti-tumor activity in non-small cell lung cancer *in vitro* and *in vivo*. Oncotarget.

[36] Chen W, Zou P, Zhao Z, Weng Q, Chen X, Ying S, Ye Q, Wang Z, Ji J, Liang G (2016). Selective killing of gastric cancer cells by a small molecule via targeting TrxR1 and ROS-mediated ER stress activation. Oncotarget.

[37] Gorrini C, Harris IS, Mak TW (2013). Modulation of oxidative stress as an anticancer strategy. Nat Rev Drug Discov.

[38] Saini V, Manral A, Arora R, Meena P, Gusain S, Saluja D, Tiwari M (2017). Novel synthetic analogs of diallyl disulfide triggers cell cycle arrest and apoptosis via ROS generation in MIA PaCa-2 cells. Pharmacol Rep.

[39] Malumbres M, Barbacid M (2005). Mammalian cyclin-dependent kinases. Trends Biochem Sci.

[40] Zhang Z, Wang H, Li M, Agrawal S, Chen X, Zhang R (2004). MDM2 is a negative regulator of p21WAF1/CIP1, independent of p53. J Biol Chem.

[41] Banerjee A, Banerjee V, Czinn S, Blanchard T (2017). Increased reactive oxygen species levels cause ER stress and cytotoxicity in andrographolide treated colon cancer cells. Oncotarget.

[42] Wang Q, Wang H, Jia Y, Pan H, Ding H (2017). Luteolin induces apoptosis by ROS/ER stress and mitochondrial dysfunction in gliomablastoma. Cancer Chemother Pharmacol.

[43] Fulda S, Gorman AM, Hori O, Samali A (2010). Cellular stress responses: cell survival and cell death. Int J Cell Biol.

[44] Harding HP, Zhang Y, Ron D (1999). Protein translation and folding are coupled by an endoplasmic-reticulum-resident kinase. Nature.

[45] Shi Y, Vattem KM, Sood R, An J, Liang J, Stramm L, Wek RC (1998). Identification and characterization of pancreatic eukaryotic initiation factor 2 alpha-subunit kinase, PEK, involved in translational control. Mol Cell Biol.

[46] Zhang K, Kaufman RJ (2008). From endoplasmic-reticulum stress to the inflammatory response. Nature.

[47] Lee ES, Yoon CH, Kim YS, Bae YS (2007). Thedouble-strand RNA dependent protein kinase PKR plays a significant role in a sustained ER stress induced apoptosis. FEBS Lett.

[48] Liu Y, Gong W, Yang ZY, Zhou XS, Gong C, Zhang TR, Wei X, Ma D, Ye F, Gao QL (2017). Quercetin induces protective autophagy and apoptosis through ER stress via the p-STAT3/Bcl-2 axis in ovarian cancer. Apoptosis.

[49] Bromberg JF, Wrzeszczynska MH, Devgan G, Zhao Y, Pestell RG, Albanese C, Darnell JE (1999). Stat3 as an oncogene. Cell.

[50] Tumang JR, Hsia CY, Tian W, Bromberg JF, Liou HC (2002). IL-6 rescues the hypo responsiveness of c-Rel deficient B cells independent of Bcl-xL, Mcl-1, and Bcl-2. Cell Immunol.

[51] Feng C, Xia Y, Zou P, Shen M, Hu J, Ying S, Pan J, Liu Z, Dai X, Zhuge W, Liang G, Ruan Y (2017). Curcumin analog L48H37 induces apoptosis through ROS-mediated endoplasmic reticulum stress and STAT3 pathways in human lung cancer cells. Mol Carcinog.

[52] Alexandrow MG, Song LJ, Altiok S, Gray J, Haura EB, Kumar NB (2012). Curcumin: a novel Stat3 pathway inhibitor for chemoprevention of lung cancer. Eur J Cancer Prev.

[53] Liu L, Liu YL, Liu GX, Chen X, Yang K, Yang YX, Xie Q, Gan HK, Huang XL, Gan HT (2013). Curcumin ameliorates dextran sulfate sodium-induced experimental colitis by blocking STAT3 signaling pathway. Int Immunopharmacol.

