# Supplementary material for: Novel allylated monocarbonyl analogs of curcumin induce mitotic arrest and apoptosis by reactive oxygen species-mediated endoplasmic reticulum stress and inhibition of STAT3
Source: Oncotarget. 2017 Sep 15;8(60):101112–29. doi: 10.18632/oncotarget.20924 (PMC5731860; doi:10.18632/oncotarget.20924)
Supplement: Supplementary file 1 [file oncotarget-08-101112-s001.pdf]

Novel allylated monocarbonyl analogs of curcumin induce mitotic arrest and apoptosis by reactive oxygen species-mediated endoplasmic reticulum stress and inhibition of STAT3

SUPPLEMENTARY MATERIALS

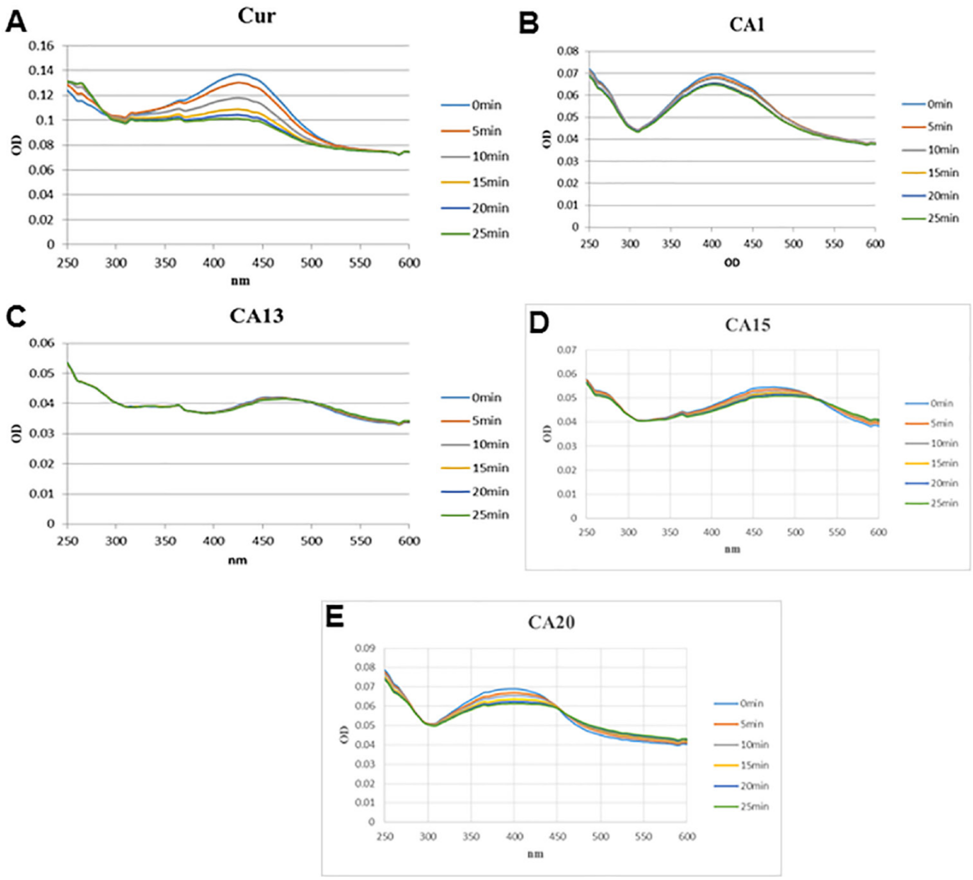

Supplementary Figure 1: Illustrates the stability of selective allylated MAC in PBS.

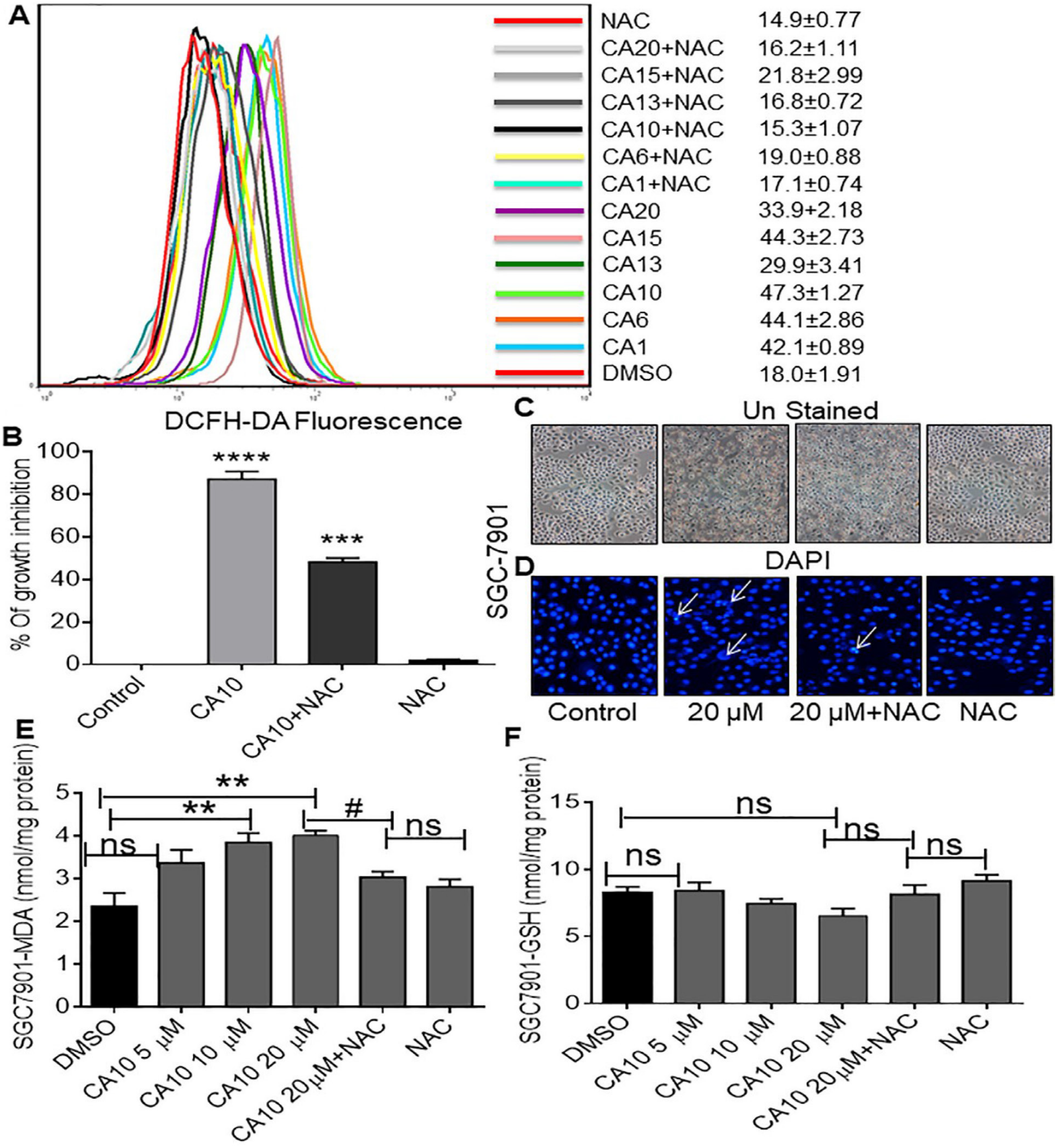

Supplementary Figure 2: (**A**) Indicates CA10 mediated ROS accumulation in SGC-7901 cells. (**B**) Graph illustrating the CA10 mediated growth inhibition in SGC-7901 with NAC pretreatment ($^{***}P < 0.001$, $^{****}P < 0.0001$ compared to the control group). (**C** and **D**) Indicates the CA10 mediated morphological feature of apoptotic cell death in SGC-7901 (cell shrinkage, loss of membrane integrity, nuclear swelling and condensed chromatin). (**E** and **F**) Illustrates the CA10 mediated imbalance in the oxidative stress marks such as MDA and total GSH ($^{**}P < 0.01$, compared to the control group $^{\#}P < 0.05$ compared to the treatment group (CA10 20 μM) and 'ns' indicates no significant difference. There was no significant changes in the level total GSH.
